# Prevalence of tuberculosis and treatment outcome among university students in Northwest Ethiopia: a retrospective study

**DOI:** 10.1186/s12889-015-1378-1

**Published:** 2015-01-21

**Authors:** Beyene Moges, Bemnet Amare, Gizachew Yismaw, Meseret Workineh, Shitaye Alemu, Desalew Mekonnen, Ermias Diro, Belay Tesema, Afework Kassu

**Affiliations:** Department of Immunology and Molecular Biology, School of Biomedical and Laboratory Sciences, College of Medicine and Health Sciences, University of Gondar, P.O. BOX 196, Gondar, Ethiopia; Department of Biochemistry, College of Medicine and Health Sciences, University of Gondar, Gondar, Ethiopia; Department of Microbiology, School of Biomedical and Laboratory Sciences, College of Medicine and Health Sciences, University of Gondar, Gondar, Ethiopia; Department of Internal Medicine, College of Medicine and Health Sciences, University of Gondar, Gondar, Ethiopia

**Keywords:** Tuberculosis, Students, Congregated setting, Ethiopia

## Abstract

**Background:**

Universities tend to be highly congregate settings, both in the classroom and in residences, and thus provide special opportunities for large number of persons to be exposed to a person with tuberculosis (TB). Despite the high prevalence of TB in Ethiopia, the TB prevalence and the treatment outcome among students have never been studied. Therefore, this study was aimed at determining the prevalence and treatment outcome of TB among students at University of Gondar from January 2007 to December 2011.

**Methods:**

Data on age, sex, TB type, category, and treatment outcome of students with TB was collected from medical records of University of Gondar Hospital, TB Directly Observed Treatment Short Course (DOTS) clinic. All TB cases diagnosed with smear, culture, and/or radiography were included in the study.

**Results:**

During the five year study period in the university, there were an average of 36 students with TB per year out of a mean of 10,036 enrolled students. Smear positive pulmonary TB, smear negative pulmonary TB, and extra pulmonary TB, respectively, were observed in 46 (25.4%), 81 (44.8%) and 54 (29.8%) of the cases. The prevalence of all forms of TB per 100,000 populations in the University ranged from 297.6 in 2009 to 404 in 2011, respectively. The prevalence of TB in the Social Sciences and Humanities Faculty was higher than the one observed in the Medical College. The overall treatment outcome was classified as cured in 36 (19.9%), completed in 91 (50.3%), defaulted in 9 (5%), failed in 3 (1.7%), died in 1 (0.6%), and transferred out in 41 (22.7%) of the cases. Treatment success rate (TSR) among students in University was generally low ranging from 58.1% in 2009 to 82.9% in 2011 with a mean TSR of 70.2%.

**Conclusion:**

The prevalence of TB is higher in comparison to the national figure among students in University of Gondar. Active surveillance systems could be important to get a clear picture of the TB situation in such settings. Assessing the factors associated with the high prevalence to gear the TB control strategy could also be essential.

## Background

Boarding schools like colleges are one of the congregated settings that create favorable conditions for TB transmission [1,2]. The crowded situation both in classrooms and dormitories increases the risk of exposure to TB and other aerosol infections. While the probability of transmission between individuals depends upon many factors, it greatly increases if TB becomes active and remains untreated; a person with active but untreated TB can infect 10–15 people per year [3]. Thus such kinds of congregated settings should be target to implement TB infection control measures.

The prevalence of TB among college students in some countries like China is reported to be high [4] and there were several reported TB outbreaks in Chinese colleges [5,6] and elsewhere [5,6]. Several other studies also revealed high risk of acquiring TB infection and subsequent transmission among college students [7–18]. A study conducted among students of Jimma University, southwest Ethiopia, showed that contact in the campus to students who have TB seems to be one of the major factors responsible for the acquisition of TB [19]. As to our knowledge, despite the fact that Ethiopia rank 7th out of the 22 high TB burden countries [20], the TB prevalence and the treatment outcome among students in the study area have never been studied. Therefore, this study was aimed at determining the prevalence and treatment outcome of TB among students at University of Gondar (UoG) in northwest Ethiopia, from January 2007 to December 2011.

## Methods

### Study setting

The University of Gondar is one of the 31 universities in Ethiopia. Currently, there are more than twenty thousand students enrolled in this University. The University has five main campuses: College of Medicine and Health Sciences (CMHS) campus, Maraki Campus, Atse Theodros Campus, Atse Fasil Campus and Meles Zenawi Campus. The total number of students enrolled in the university during the respective years from 2007 to 2011 were 8817, 9109, 10418, 10453, and 11385 (Table 1). Table 1 summarizes the total numbers of students in each faculty or college during the five years period. CMHS is a medical college enrolling students of different categories of health sciences, medicine and pharmacy in the regular and extension programs. The college has a tertiary hospital, the University of Gondar Hospital, which renders clinical service for about six million people in northwest Ethiopia. Students attach at different wards and outpatient departments of the hospital during their clinical courses. The Maraki campus which is 3 km away from CMHS encompasses Faculty of Social Sciences and Humanities, School of Law, and School of Education. The third campus is Atse Theodros campus which is named after Emperor Theodros 4^th^ of Ethiopia. It is located near Maraki campus and it hosts the head office of the University. It encompasses the Faculty of Veterinary Medicine, Faculty of Natural and Computational Sciences, Faculty of Agriculture and Faculty of Business and Economics. The fourth campus is the newly opened Atse Fasil campus which is named after Emperor Fasiledes of Ethiopia. It encompasses the School of Technology. Our study, however, didn’t include students from this campus as it is newly launched. The fifth campus is Meles Zenawi campus which is named after the late Prime Minster Meles Zenawi of Ethiopia. Our study didn’t also include students from this campus as students are to be transferred yet.

**Table 1:** **The total number of students registered per campus over the five year study period**

**Campus**	**2007**	**2008**	**2009**	**2010**	**2011**
**CMHS**	2919	3194	2705	2855	2729
**Maraki**	1530	1811	2901	2555	3059
**Atse Theodros**	4368	4104	4812	5043	5597
**Total**	**8817**	**9109**	**10418**	**10453**	**11385**

CMHS = College of Medicine and Health Sciences.

On average, a dormitory is shared by six to eight students in all the campuses. However, the numbers of students in a dormitory may reach up to 20–30 in some situations. While 100–200 students share a class room 2000–5000 students share a single dining hall three times a day. Though there are student clinics in each campus of the University, all students get free medical service at the University of Gondar Hospital.

### Data collection procedure

Using a standardized form data on age, sex, TB type, category, and treatment outcome of students with TB was collected from medical records of University of Gondar Hospital, Directly Observed Treatment Short Course (DOTS) clinic. All TB cases diagnosed with smear, culture, and/or radiography were included in the study. All students of University of Gondar who were diagnosed and initiated on TB treatment at the Hospital DOTS clinic between January 2007 and December 2011 were considered except those with incomplete information. Those cases that were under ongoing TB treatment were also excluded. Data was collected by the physician investigators and nurses working in the DOTS clinic.

### Definitions

Clinical case and treatment outcome definitions for TB were used according to the standard definitions of the National Tuberculosis and Leprosy Control Program guideline (NTLCP) adopted from WHO [21].

### Treatment success rate (TSR)

It is the sum of the percentages of cure and treatment completed rounded off to the nearest digit [22].

### Data analysis

Data was grouped based on the year of diagnosis and was analyzed using statistical package for social sciences (SPSS) version 16 and descriptive statistics was carried out.

### Ethical considerations

The study was conducted after ethical approval was obtained from Institutional Review Board of the University of Gondar. Permission of access to participants’ data was also obtained from the University of Gondar Hospital. No patient identifiers were used during data collection.

## Results

There were a total of 181 students diagnosed for TB at University of Gondar Hospital from January 2007 to December 2011. Majority (149, 82.3%) of them were males. The mean (±SD, range) age of the students was 22.7 (±1.54, 17–28) years. Majority (166, 91.7%) of the cases were between 19 and 23 years of age. Generally, most of the TB cases were from Maraki campus (83, 45.8%) followed by CMHS (68, 37.6%) and Atse Theodros campuses (30, 16.6%). The trend and distribution of all forms of TB per campus is shown in Table 2.

**Table 2:** **Trend of all forms of TB by campus among students in University of Gondar, 2007–2011, N = 181**

**Year**	**Campus**			
	**Maraki N (%)**	**CMHS N (%)**	**Atse Theodros N (%)**	**Total N (%)**
**2007**	28 (84.8)	5 (15.2)	0 (0)	33 (100)
**2008**	18 (56.2)	13 (40.6)	1 (3.1)	32 (100)
**2009**	11 (35.5)	11 (35.5)	9 (29)	31 (100)
**2010**	10 (25.6)	21 (53.8)	8 (20.5)	39 (100)
**2011**	16 (34.8)	18 (39.1)	12 (26.1)	46 (100)
**Total**	83 (45.9)	68 (37.6)	30 (16.6)	181 (100)

TB = Tuberculosis, N = number, CMHS = College of Medicine and Health Sciences.

### Prevalence and category of tuberculosis

Prevalence of all forms of TB per 100,000 populations in the University ranged from 297.6 in 2009 to 404 in 2011, respectively. The prevalence of all forms of TB per 100,000 populations in Maraki Campus ranged from 379.2 to 1830.1 while it was between 171.3 and 735.6 in CMHS and between 0 and 214.4 in Atse Theodros Campuses. The prevalence showed decrement from 347.3/100,000 populations in 2007 to 297.6/100,000 in 2009 with a subsequent rise to 373.1 and 404/100,000 populations in 2010 and 2011 respectively. The highest campus specific TB prevalence (1830.1/100,000 populations) detected was from Maraki in 2007 while the lowest was in Atse Theodros where the prevalence of TB was zero in the same year. The TB prevalence in Maraki campus in the years 2007 and 2008 was 3.2 and 2.3 times higher than the prevalence in the general population, respectively. Table 3 compares the TB prevalence of each campus and the University to that of the respective national prevalences.

**Table 3:** **Prevalence of all forms of TB among students in the University of Gondar in comparison to the national TB prevalence, 2007-2011**

**Year**	**Prevalence of TB per 100,000 populations in Maraki**	**Prevalence of TB per 100,000 populations in CMHS**	**Prevalence of TB per 100,000 populations in Atse Theodros**	**Prevalence of TB per 100,000 populations in UOG**	**National prevalence of TB per 100,000 populations**
**2007**	1830.1	171.3	0	347.3	579^a^
**2008**	993.9	407	24.4	351.3	432^b^
**2009**	379.2	406.7	187	297.6	406^c^
**2010**	391.4	735.6	158.6	373.1	394^d^
**2011**	523.1	659.6	214.4	404	273^e^

TB = Tuberculosis, CMHS = College of Medicine and Health Sciences, UOG = University of Gondar.

^a^WHO 2008, ^b^WHO 2009, ^c^WHO 2010, ^d^WHO 2011, ^e^WHO 2012.

TB cases were categorized in to new, relapse, failure, default, transferred in and other cases before treatment was begun in all the five years. Majority (140, 77.3%) of them were new cases. However, there were two relapse, two failure and two defaulter cases seen during the five years period.

TB type was classified as smear negative pulmonary TB in 81 (44.8%), extra pulmonary TB in 54 (29.8%) of the cases and smear positive pulmonary TB in 46 (25.4%). The proportion of smear positive pulmonary TB showed decrement from 45.5% in 2007 to 9.7% in 2009 with a subsequent rise to 17.9% and 28.3% in 2010 and 2011, respectively. The trend and distribution of TB types at the University of Gondar during the five years period is described in Table 4.

**Table 4:** **Trend of TB type among students in the University of Gondar, 2007–2011, N = 181**

**Year**	**TB type**			
	**SP N (%)**	**SN N (%)**	**EP N (%)**	**Total N (%)**
**2007**	15 (45.5)	7 (21.2)	11 (33.3)	33 (100)
**2008**	8 (25)	16 (50)	8 (25)	32 (100)
**2009**	3 (9.7)	19 (61.3)	9 (29)	31 (100)
**2010**	7 (17.9)	21 (53.8)	11 (28.2)	39 (100)
**2011**	13 (28.3)	18 (39.1)	15 (32.6)	46 (100)
**Total**	46 (25.4)	81 (44.6)	54 (29.8)	181 (100)

TB = Tuberculosis, N = Number, SP = Smear positive pulmonary tuberculosis, SN = Smear negative pulmonary tuberculosis, EP = Extra-pulmonary tuberculosis.

### Treatment outcome

The overall treatment outcome was classified as cured in 36 (19.9%), completed in 91 (50.3%), defaulted in 9 (5%), failed in 3 (1.7%), died in 1 (0.6%), and transferred out in 41 (22.7%) of the cases. The rate of cure among the smear positive pulmonary TB cases showed a dramatic fall from 21.2% in 2007 to 6.5% in 2009 with a subsequent rise to 41.3% in 2011. Failure was reported in three of the five years, 2007, 2008 and 2010 while default was reported in all the five years except in 2007. One case of treatment failure case was reported in each of the following years, 2007, 2008 and 2010. The rate of default ranged from 0% in 2007 to 10.3% in 2010 and the trend was increasing except in the year 2011 (6.5%). A single case of death was documented in one of the five years, 2010 [Table 5]. The treatment success rate (TSR) among the students in University of Gondar was generally low ranging from 58.1% in 2009 to 82.6% in 2011 with a mean TSR of 70.2%. In comparison to the national TSR, the students’ TSR was lower in all the five years. The trend of TB treatment outcome, TSR among the students in the University of Gondar, and the national TSR for comparison is showed in Table 5.

**Table 5:** **Trend of TB treatment outcome and TSR among students in University of Gondar in comparison to the national TSR, 2007–2011, N = 181**

**Year**	**Treatment outcome**						**TSR (%)**	
	**TSN (%)**	**TO N (%)**	**F N (%)**	**D N (%)**	**Died N (%)**	**Total N (%)**	**TSR in UoG**	**TSR in Ethiopia**
**2007**	22 (66.7)	10 (30.3)	1 (3)	0 (0)	0 (0)	33 (100)	66.7	84^a^
**2008**	26 (81.2)	4 (12.5)	1 (3.1)	1 (3.1)	0 (0)	32 (100)	81.2	84^b^
**2009**	18 (58.1)	12 (35.7)	0 (0)	1 (3.2)	0 (1)	31 (100)	58.1	84^c^
**2010**	23 (59)	10 (25.6)	1 (2.6)	4 (10.3)	1 (2.6)	38 (100)	59	84^d^
**2011**	38 (82.6)	5 (10.9)	0 (0)	3 (6.5)	0 (0)	46 (100)	82.6	83^e^
**Total**	127 (70.1)	41 (22.6)	3 (1.7)	9 (5)	1 (0.6)	181 (100)		

TB = Tuberculosis, TSR = Treatment success rate, N = Number, TS = Treatment success (Completed and cured), TO = Transferred out, F = Failure, D = Defaulted.

^a^WHO 2008, ^b^WHO 2009, ^c^WHO 2010, ^d^WHO 2011, ^e^WHO 2012.

## Discussion

Colleges tend to be highly congregated settings, both in classrooms and dormitories, and thus provide special opportunities for a lot of people to be exposed to a person with TB [1,2]. This study, which is the first in its type in northwest Ethiopia, revealed high prevalence of TB per 100,000 populations among students in University of Gondar (Table 3). High prevalence of TB among college students have been a growing concern in several other countries [23,24]. The prevalence in the University was increasing since 2009 and become even higher than the national prevalence in the year 2011 [25].

This study revealed that campus specific prevalence was higher in some of the years in comparison to the respective national prevalence [20,25–28]. For instance, the prevalence in Maraki during 2007 and 2008 was 3.2× [26] and 2.3× [27] higher than the respective national prevalence during the same years. Likewise the prevalence in CMHS during 2010 and 2011 was 2.4× [20]) and 1.9× [5] higher than the national prevalence during the respective years (Table 3). Of note, the national TB prevalence during these years was not adjusted for age making the comparison difficult with participants of the current study as they all were between 17 and 28 years of age.

An increasing trend of TB prevalence was observed in Atse Theodros Campus from zero in 2007 to 214.4/100,000 populations in 2011. The very low TB prevalences (0 and 24.4/100,000 populations) in the years 2007 and 2008 among students in Atse Theodros campus could be explained by the fact that the campus was established at those times and students from that campus might have registered as from Maraki; the two campuses are a walking distance apart. Likewise, the high prevalence of TB in Maraki campus during 2007 and 2008 could be for the same reason; students from Atse Theodros campus might have registered as if they were from Maraki while only the students from Maraki were used as denominator. The increasing TB trend in Atse Theodros campus in the subsequent years (Tables 1 and 2) could be from proper documentation of the campus addresses. It could also be from an increment of the actual TB prevalence. An increasing trend was also observed in CMHS between 2007 and 2011.

One of the reasons for the high and increasing prevalence of TB in the University could be overcrowding [1,2]. Low level of knowledge and attitude about the TB transmission and prevention mechanisms could be the other factors [29–31]. Poor TB infection control activities in the campuses, especially in Maraki, may be another explanation. Occupational exposure could also be a factor especially to those in CMHS as medical and health science students stay in outpatient and inpatient departments of University of Gondar Hospital during their clinical attachments. Even though the current study wasn’t aimed at assessing the incidence of TB, similar observation has been cited in India by which substantially higher annual risk of TB infection among young nursing trainees than in the general Indian population [9]. The reasons why TB prevalence was high in Maraki than CMHS where students are exposed to TB patients everyday requires further investigation. Besides, the high prevalence of TB in the study area could pose problems to the TB control in the general population as TB from universities may spread through students and staff into the community. The probability of transmission between students greatly increases as a significant proportion of the TB cases (25.4%) were smear positive. A student with active and untreated TB could infect 10–15 other students per year [3] and further increase the rate of transmission. Periodic screening and characterization of associated factors for transmission with subsequent intervention in the University could impact on the TB control in the community.

This study found out that there was a relatively lower TSR among students in the University of Gondar in comparison to the respective national figures [20,25–28] in all the years except 2008 and 2011 (Table 5). The lower TSR could be due to the relatively high transferred out rate. There were defaulters and failures as well. Failure was reported in three of the five years while default was reported in all the five years except 2007 (Table 5). There was also a case that died while on retreatment. This group of students had substantially increased risk of developing MDR-TB (one was confirmed MDR-TB case) urging the need to strengthen the TB control program in colleges and other higher education systems in the country.

WHO recommends, based on expert opinion, an empirical five drug retreatment regimen after failing, interrupting, or relapsing from prior treatment in low and middle-income countries [32,33]. Likewise, the six students at UOG with treatment failure, relapse and default were started with the empirical retreatment regimen. However, the recently updated WHO guideline recommends drug-susceptibility testing (DST) prior to retreatment [34] and treatment of confirmed treatment failures with region-specific standardized regimens [35] In contrary to the aforementioned WHO recommendations, none of the six students who were on retreatment regimen underwent DST prior to start of therapy. This might mainly be due to the poor access for DST services in Ethiopia. Nevertheless, it is known inadequate regimens amplify drug resistance [36–39] with resultant grave treatment outcomes. This is evidenced by the death of one of the relapse cases and development of MDR-TB by one of the treatment failure cases while on the standard retreatment regimen. This alarms the urgent need to further scale up the TB culture and DST laboratory capacity building in Ethiopia with simultaneous revision of current retreatment guidelines based on available national data. Further studies are also required to ease decision making for policy makers on TB prevention and control strategies in congregated settings like colleges.

There was high transferred out rate (22.7%) during the five years among students in the University which could probably be aggravated by winter and summer vacations. The transferred out cases might have been cured or successfully completed. It could also result in to unfavorable TB treatment outcomes like treatment failure and default unless adequate information was given to the students. The lack of information about transferred out cases, therefore, limits the strength of reports about TSR from DOTS clinics. Lack of clear guideline between referring and accepting DOTS clinics requires evaluation and immediate intervention. The primary DOTS clinic should have collected treatment outcome data from the accepting clinics. Therefore, efficient counseling and follow up of transferred out patients is mandatory to avoid poor treatment outcomes. Similarly, developing a clear guideline on how to trace and report transferred out cases is essential. All in all, a strong contact tracing system should be implemented in such settings to avoid spill over of TB and MDR-TB.

In summary, there was high prevalence of TB among students in the University. TB treatment outcome was also far from the respective national TSR in most of the years. Risk of developing MDR-TB was also a problem in retreatment cases. Therefore, strong TB prevention and control programs are required at the University and other universities in Ethiopia. Use of DST prior to initiation of retreatment regimen should also be stressed for treatment failure, relapse and default cases especially in congregated settings as the risk of MDR-TB transmission could be enhanced. Because of their exposure to large numbers of smear-positive TB cases managed at hospitals, health care workers and students in high TB burden settings, like the current study setting, are at higher risk of developing latent TB infection as compared with the general population. This indicates the importance of testing new students for TB and periodic testing for Latent TB infection in order to prevent further spread of TB. We recommend that universities should look and act on factors which facilitate the development of TB disease like HIV/AIDS and malnutrition.

This is the first organized attempt to report on the situation of TB in a university in Ethiopia. It is an eye opener to researchers and policy makers in the country as TB in health care settings and universities seems neglected. The limitations of this study arose mainly because it is a retrospective study. Analysis was completely reliant on the data from DOTS clinic registration book and patient charts. It couldn’t be supported by active interviews and focus group discussions. The year of study of the students would be interesting to have but that information wasn’t available neither in the registration book nor in patient charts.

## Conclusion

There is high prevalence of TB among students in University of Gondar which demands urgent intervention. Campus specific prevalence was even higher in Maraki and CMHS demanding further research and strengthening TB infection control systems. Use of DST prior to initiation of retreatment regimen should also be stressed for treatment failure, relapse and default cases especially in congregated settings as the risk of MDR-TB transmission could be enhanced. Besides, periodic and active TB surveillance systems are required in such group of people to get a clear picture of the TB situation. Assessing the factors associated with the high TB prevalence and poor treatment outcomes among students could also be imperative to gear the TB control strategy.

## Abbreviations

CMHS: College of Medicine and Health Sciences
DOTS: Directly observed treatment short course
DST: Drug susceptibility testing
MDR: Multidrug resistant
MDR-TB: Multidrug resistant Tuberculosis
NTLCP: National Tuberculosis and Leprosy Control Program guideline
SD: Standard deviation
SPSS: Statistical Package for Social Sciences
TB: Tuberculosis
TSR: Treatment success rate
UoG: University of Gondar
WHO: World Health Organization

## References

[1] The Lodi Tuberculosis Working Group (1994). A school- and community-based outbreak of Mycobacterium tuberculosis in northern Italy, 1992–3. Epidemiol Infect.

[2] Ridzon R, Kent JH, Valway S, Weismuller P, Maxwell R, Elcock M (1997). Outbreak of drugresistant tuberculosis with second-generation transmission in a high school in California. J Pediatr.

[3] World Health Organization (2007). Tuberculosis Fact Sheet No104-Global and Regional Incidence.

[4] Hong F, Tu DH, An YS, Pan LN (2008). Reform and practice of tuberculosis control in Beijing. J Chinese Antituberculosis Assoc.

[5] Zhou XS (2006). Analysis of outbreak of tuberculosis in university. J Clin Pulm Med.

[6] Ge JR, Yuan CY, Ma LX, Zhang JC, Ma WS, Guo FA (2006). Analysis epidemic of tuberculosis in colleges of Shijiazhuang. Health Med Res Practice Higher Institutions.

[7] Stein-Zamir C, Volovik I, Rishpon S, Atamna A, Lavy A, Weiler-Ravell D (2006). Tuberculosis outbreak among students in a boarding school. Eur Respir J.

[8] Maciel EL, Meireles W, Silva AP, Fiorotti K, Dietze R (2007). Nosocomial Mycobacterium tuberculosis transmission among healthcare students in a high incidence region, in Vitoria, State of Espirito Santo. Rev Soc Bras Med Trop.

[9] Christopher DJ, James P, Daley P, Armstrong L, Isaac BTJ, Thangakunam B (2011). High annual risk of tuberculosis infection among nursing students in South India: a cohort study. PLoS One.

[10] Zinov’ev IP, Pozdeeva NV (2008). Study of a risk for tuberculosis among medical students. Probl Tuberk Bolezn Legk.

[11] Zinov’ev IP, Pozdeeva NV (2007). Medical students as an independent tuberculosis-risk group. Probl Tuberk Bolezn Legk.

[12] Teixeira EG, Kritski A, Ruffino Netto A, Steffen R, Silva JR LE, Belo M (2010). Medical students at risk of nosocomial tuberculosis. J Hosp Infect.

[13] Munoz Sanchez AI, Bertolozzi MR (2011). Functioning of the concept of vulnerability to tuberculosis amongst university students. Cien Saude Colet.

[14] Murad MA, Abdulmageed SS (2010). Tuberculosis screening among health sciences students in Saudi Arabia in 2010. Ann Saudi Med.

[15] Mussi TV, Traldi MC, Talarico JN (2012). Knowledge as a factor in vulnerability to tuberculosis among nursing students and professionals. Rev Esc Enferm USP.

[16] Li X, Zhang S, Yan H, Zhang T, Zhang J (2010). Barriers to tuberculosis control and prevention in undergraduates in Xi’an, China: a qualitative study. J Public Health Policy.

[17] Hohmuth BA, Yamanija JC, Dayal AS, Nardell E, Salazar JJ, Smith Fawzi MC (2006). Latent tuberculosis infection: risks to health care students at a hospital in Lima. Peru Int J Tuberc Lung Dis.

[18] Guo JL, Xu Q, Liu YQ (2006). Investigation on freshmen’s tuberculosis infection in 33 universities in Beijing. Zhonghua Yu Fang Yi Xue Za Zhi.

[19] Lissane Seifu, Sileshi T/Mariam. Tuberculosis among Students of Jimma University. Ethiop J Health Sci , 2001, 11 (1): 47–51

[20] World Health Organization. Global Tuberculosis Control. Country Profile: Ethiopia Available at: http://www.who.int/tb/publications/global_report/2011/pdf/eth.pdf.

[21] Ministry of Health of Ethiopia (MOH). Tuberculosis, Leprosy and TB/HIV Prevention and Control Programme Manual. Addis Ababa: MOH 4th edition. 2008, Treatment of tuberculosis guidelines. 4th ed. Geneva: World Health Organization (WHO/HTM/STB/2009.420).); 2010.

[22] World Health Organization. Tuberculosis Control. Country Profile: Ethiopia Available at: http://www.who.int/tb/publications/global_report/2001/pdf/eth.pdf.

[23] Zhang SR, Yan H, Zhang JJ, Zhang TH, Li XH, Zhang YP (2010). The experience of college students with pulmonary tuberculosis in Shaanxi, China: a qualitative study. BMC Infect Dis.

[24] Christopher DJ, James P, Daley P, Armstrong L, Isaac BTJ, Thangakunam B (2011). High annual risk of tuberculosis infection among nursing students in south India: a cohort study. PLoS One.

[25] World Health Organization. Global tuberculosis report Geneva, Switzerland: WHO 2012 Country Profile: Ethiopia Available at: http://www.who.int/tb/publications/global_report/2012/pdf/eth.pdf.

[26] World Health Organization. Global Tuberculosis Control. WHO/HTM/TB/2008.393), Country Profile: Ethiopia Available at: http://www.who.int/tb/publications/global_report/2008/pdf/eth.pdf.

[27] World Health Organization. Global Tuberculosis Control. Country Profile: Ethiopia. Available at: http://www.who.int/tb/publications/global_report/2009/pdf/eth.pdf.

[28] World Health Organization. Global Tuberculosis Control: Epidemiology, Strategy, fi Nancing. WHO Report 2009. WHO/ HTM/TB/2009.411. Geneva, Switzerland: WHO; 2010.

[29] Ahmed E, Ibrahim A, Mulualem A, Adinew D, Zelalem Y, Kassu D (2013). Assessment of patients’ knowledge, attitude, and practice regarding pulmonary tuberculosis in Eastern Amhara Regional State, Ethiopia: cross-sectional study. Am J Trop Med Hyg.

[30] Bati J, Legesse M, Medhin G (2013). Community’s knowledge, attitudes and practices about tuberculosis in itang special district, gambella region, south western Ethiopia. BMC Public Health.

[31] Rahel A, Meaza D. Assessment of knowledge and practices related to tuberculosis and associated factors among HIV. GJMEDPH. 2012;1(2):35–42. Positive People In Addis Ababa, Ethiopia.

[32] World Health Organization (2006). 2006 Guidelines for the Programmatic Management of Drug-Resistant Tuberculosis.

[33] World Health Organization (2009). 2009 Treatment of Tuberculosis: Guidelines – 4th Edition.

[34] World Health Organization (2009). 2009 Global Tuberculosis Control - a Short Update to the 2009 Report.

[35] World Health Organization. Treatment of Tuberculosis: Guidelines – 4th ed. WHO/HTM/TB/2009.420. Geneva, Switzerland: WHO; 2010. 23741786

[36] Menzies D, Benedetti A, Paydar A, Royce S, Pai M, Burman W, et al. Standardized treatment of active tuberculosis in patients with previous treatment and/or with mono-esistance to isoniazid: a systematic review and meta-analysis. PLoS Med. 2009;6:e1000150. doi:10.1371/journal.pmed.1000150. 10.1371/journal.pmed.1000150 PMC2736403 20101802

[37] Espinal MA (2003). Time to abandon the standard retreatment regimen with first-line drugs for failures of standard treatment. Int J Tuberc Lung Dis.

[38] Matthys F, Rigouts L, Sizaire V, Vezhnina N, Lecoq M, Golubeva V (2009). Outcomes after chemotherapy with WHO category II regimen in a population with high prevalence of drug resistant tuberculosis. PLoS One.

[39] Quy HT, Lan NT, Borgdorff MW, Grosset J, Linh PD, Tung LB (2003). Drug resistance among failure and relapse cases of tuberculosis: is the standard retreatment regimen adequate?. Int J Tuberc Lung Dis.

